# Supplementary material for: Different Prognostic Values of Tumour and Nodal Response to Neoadjuvant Chemotherapy Depending on Subtypes of Inflammatory Breast Cancer, a 317 Patient-Study
Source: Cancers (Basel). 2022 Aug 15;14(16):3928. doi: 10.3390/cancers14163928 (PMC9406352; doi:10.3390/cancers14163928)
Supplement: Supplementary file 1 [file cancers-14-03928-s001.zip › cancers-1837701-supplementary.pdf]

Supplementary Materials

Table of contents

Table S1: Details of Sataloff's classification 2

Table S2: Details of excluded patients (no neoadjuvant chemotherapy and/or HR/HER2 status not available and/or no surgery) 2

Table S3: Baseline demographics, clinical characteristics, and response after neoadjuvant chemotherapy for HR+ and/or HER2+ tumours 3

Table S4: Chemotherapy, surgery, and radiotherapy details for HR+ and/or HER2+ tumours 4

Table S5: Univariate analysis of prognostic factors for overall survival (OS) stratified by triple-negative status 5

Table S6: Multivariate analysis of prognostic factors for overall survival (OS) stratified by triple-negative status 6

Figure S1: Flow chart of the patient population 7

Figure S2: Flowchart of different therapeutic strategies..8

Figure S3: Association between DFS and Sataloff response by triple-negative status 9

Figure S4: Association between OS, DMFS, LRFS, and Sataloff response by triple-negative status 10

Table S1. Details of Sataloff's classification.

**Tumour A**	Total or near total therapeutic effect
**Tumour B**	More than 50% therapeutic effect but less than total or near total
**Tumour C**	Less than 50% therapeutic effect but visible effect
**Tumour D**	No therapeutic effect
**Node A**	No nodal metastasis and evidence of therapeutic effect
**Node B**	No nodal metastasis or therapeutic effect
**Node C**	Nodal metastasis present with evidence of therapeutic effect
**Node D**	Nodal metastasis present without evidence of therapeutic effect

Table S2. Details of excluded patients (no neoadjuvant chemotherapy and/or HR/HER2 status not available and/or no surgery).

	**N = 47**
Age, years	66.7 (48.6–83.8)
Age, years (category)	
<40	6/47 (12.8%)
40–49	6/47 (12.8%)
50–59	5/47 (10.6%)
≥60	30/47 (63.8%)
Body Mass Index, kg/m^2	
<25	18/46 (39.1%)
≥25 <30	13/46 (28.3%)
≥30	15/46 (32.6%)
Histology	
Ductal	39/45 (86.7%)
Lobular	3/45 (6.7%)
Other	3/45 (6.7%)
Grade SBR	
1–2	19/40 (47.5%)
3	21/40 (52.5%)
cN	
N0	11/44 (25.0%)
N+	33/44 (75.0%)
WHO performance status	
0	22/46 (47.8%)
1	20/46 (43.5%)
2–3	4/46 (8.7%)
Ki67	
<10%	0
10–30%	12/20 (60.0%)
>30%	8/20 (40.0%)
Subtype	
HR+ HER2+	2/36 (5.6%)
HR+ HER2-	16/36 (44.4%)
HR- HER2+	7/36 (19.4%)
HR- HER2-	11/36 (30.6%)

Data are median (IQR) or n/N (%). SBR = Scarff Blood Richardson. cN = clinical lymph Node. WHO = World Health Organization. HR = Hormonal Receptor. HER2 = Human Epidermoid growth factor Receptor-2.

Table S3. Baseline demographics, clinical characteristics, and response after neoadjuvant chemotherapy for HR+ and/or HER2+ tumours.

	HR+ and/or HER2+ N = 218	HR+ HER2+ N = 45	HR+ HER2- N = 127	HR- HER2+ N = 45	p Value
Age, years	53.0 (46.1–61.4)	52.9 (44.3;63.5)	51.7 (45.9;61.3)	56.1 (49.8;62.1)	0.55
Age, years (category)					0.37
<40	27/217 (12.4%)	6/45 (13.3%)	15/126 (11.9%)	6/45 (13.3%)	
40–49	58/217 (26.7%)	13/45 (28.9%)	39/126 (31%)	6/45 (13.3%)	
50–59	65/217 (30.0)	11/45 (24.4%)	35/126 (27.8%)	18/45 (40%)	
≥60	67/217 (30.9%)	15/45 (33.3%)	37/126 (29.4%)	15/45 (33.3%)	
Body Mass Index, kg/m^2					0.11
<25	61/217 (28.1%)	10/44 (22.7%)	39/127 (30.7%)	12/45 (26.7%)	
≥25 <30	76/217 (35.0%)	23/44 (52.3%)	38/127 (29.9%)	15/45 (33.3%)	
≥30	80/217 (36.9%)	11/44 (25%)	50/127 (39.4%)	18/45 (40%)	
Histology					0.28
Ductal	188/216 (87%)	39/44 (88.6%)	112/127 (88.2%)	36/44 (81.8%)	
Lobular	16/216 (7.4%)	2/44 (4.6%)	11/127 (8.7%)	3/44 (6.8%)	
Other	12/216 (5.6%)	3/44 (6.8%)	4/127 (3.2%)	5/44 (11.4%)	
Grade SBR					0.12
1–2	115/215 (53.5%)	19/44 (43.2%)	75/126 (59.5%)	21/44 (47.7%)	
3	100/215 (46.5%)	25/44 (56.8%)	51/126 (40.5%)	23/44 (52.3%)	
cN					0.55
N0	41/216 (19.0%)	10/45 (22.2%)	25/126 (19.8%)	6/44 (13.6%)	
N+	175/216 (81.0%)	35/45 (77.8%)	101/126 (80.2%)	38/44 (86.4%)	
WHO performance status					1
0	180/217 (83%)	36/45 (80%)	106/126 (84.1%)	38/45 (84.4%)	
1	35/217 (16.1%)	8/45 (17.8%)	19/126 (15.1%)	7/45 (15.6%)	
2–3	2/217 (0.9%)	1/45 (2.2%)	1/126 (0.8%)	0	
Ki67					0.66
<10%	3/121 (2.5%)	0	3/76 (4%)	0	
10–30%	55/121 (45.5%)	12/23 (52.2%)	34/76 (44.7%)	9/22 (40.9%)	
>30%	63/121 (52.1%)	11/23 (47.8%)	39/76 (51.3%)	13/22 (59.1%)	
Sataloff T					<0.0001
TA	74/216 (34.3%)	25/45 (55.6%)	18/125 (14.4%)	31/45 (68.9%)	
TB	79/216 (36.6%)	13/45 (28.9%)	57/125 (45.6%)	9/45 (20%)	
TC	50/216 (23.2%)	6/45 (13.3%)	40/125 (32%)	3/45 (6.7%)	
TD	13/216 (6.0%)	1/45 (2.2%)	10/125 (8%)	2/45 (4.4%)	
Sataloff N					<0.0001
NA	65/217 (30.0%)	23/45 (51.1%)	16/126 (12.7%)	26/45 (57.8%)	
NB	26/217 (12.0%)	8/45 (17.8%)	14/126 (11.1%)	4/45 (8.9%)	
NC	90/217 (41.5%)	14/45 (31.1%)	63/126 (50%)	12/45 (26.7%)	
ND	36/217 (16.6%)	0	33/126 (26.2%)	3/45 (6.7%)	
ypN					NC
N0	90/217 (41.5%)	31/45 (68.9%)	28/126 (22.2%)	31/45 (68.9%)	
N1	51/217 (23.5%)	10/45 (22.2%)	32/126 (25.4%)	8/45 (17.8%)	
N2	56/217 (25.8%)	4/45 (8.9%)	48/126 (38.1%)	4/45 (8.9%)	
N3	18/217 (8.3%)	0/45 (0%)	16/126 (12.7%)	2/45 (4.4%)	
Nx	2/217 (0.9%)	0/45 (0%)	2/126 (1.6%)	0/45 (0%)	
ypT					NC
ypT0	42/217 (19.4%)	10/45 (22.2%)	9/126 (7.1%)	23/45 (51.1%)	
ypTis	13/217 (6%)	7/45 (15.6%)	1/126 (0.8%)	5/45 (11.1%)	
ypT1	59/217 (27.2%)	18/45 (40%)	37/126 (29.4%)	4/45 (8.9%)	

ypT2	47/217 (21.7%)	5/45 (11.1%)	38/126 (30.2%)	3/45 (6.7%)	
ypT3	30/217 (13.8%)	4/45 (8.9%)	21/126 (16.7%)	5/45 (11.1%)	
ypT4	19/217 (8.8%)	1/45 (2.2%)	13/126 (10.3%)	5/45 (11.1%)	
ypTx	7/217 (3.2%)	0/45 (0%)	7/126 (5.6%)	0/45 (0%)	
Pathological complete response according to Sataloff	60/216 (27.8%)	22/45 (48.9%)	12/125 (9.6%)	26/45 (57.8%)	<0.0001
Pathological complete response according to ypTNM	51/218 (23.4%)	16/45 (35.6%)	9/127 (7.1%)	26/45 (57.8%)	<0.0001

Data are median (IQR) or n/N (%). Percentages may not total 100 because of rounding. *p* values were calculated using the χ^2 test, Fisher's exac*t* test or Student *t* test. HR = Hormonal Receptor. HER2 = Human Epidermoid growth factor Receptor-2. TN = Triple Negative. NC = not computed. SBR = Scarff Blood Richardson. cN = clinical lymph Node. WHO = World Health Organization. RCB = Residual Cancer Burden.

Table S4. Chemotherapy, surgery, and radiotherapy details for HR+ and/or HER2+ tumours.

	**HR+ and/or HER2+ N = 218**	**HR+ HER2+ N = 45**	**HR+ HER2- N = 127**	**HR- HER2+ N = 45**	***p* Value**
**Neoadjuvant chemotherapy protocol**	218/218 (100%)	45/45 (100%)	127/127 (100%)	45/45 (100%)	NC
Number of cycles	8 (7–8)	8 (6–8)	8 (7–8)	8 (8–8)	0.23
(F)EC-T	169/218 (77.5%)	34/45 (75.6%)	100/127 (78.7%)	34/45 (75.6%)	0.85
AC-T	24/218 (11%)	6/45 (13.3%)	14/127 (11%)	4/45 (8.9%)	0.79
Taxanes received	217/218 (99.5%)	45/45 (100%)	126/127 (99.2%)	45/45 (100%)	NC
Antracyclines received	193/218 (88.5%)	40/45 (88.9%)	114/127 (89.8%)	38/45 (84.4%)	0.63
Platinium sels received	0	0	0	0	NC
Trastuzumab alone	75/218 (34.4%)	38/45 (84.4%)	1/127 (0.8%)	35/45 (77.8%)	<0.0001
Trastuzumab + Pertuzumab	14/218 (6.4%)	6/45 (13.3%)	0/127 (0%)	8/45 (17.8%)	
**Adjuvant systemic treatment**	204/218 (93.6%)	45/45 (100%)	117/127 (92.1%)	41/45 (91.1%)	0.14
Hormonotherapy	164/218 (75.2%)	44/45 (97.8%)	117/127 (92.1%)	2/45 (4.4%)	<0.0001
Trastuzumab	85/218 (39%)	39/45 (86.7%)	5/127 (3.9%)	40/45 (88.9%)	<0.0001
Adjuvant chemotherapy	1/218 (0.5%)	0/45 (0%)	0/127 (0%)	1/45 (2.2%)	NC
TDM-1	3/218 (1.4%)	2/45 (4.4%)	0/127 (0%)	1/45 (2.2%)	NC
Capecitabine	1/218 (0.5%)	0/45 (0%)	0/127 (0%)	1/45 (2.2%)	0.15
**Surgery**	218/218 (100%)	45/45 (100%)	127/127 (100%)	45/45 (100%)	NC
Mastectomy + SLND	1/218 (0.5%)	0/45 (0%)	1/127 (0.8%)	0/45 (0%)	NC
Mastectomy + ALND	214/218 (98.2%)	45/45 (100%)	123/127 (96.9%)	45/45 (100%)	
Tumourectomy + SLND	0	0/45 (0%)	0/127 (0%)	0/45 (0%)	
Tumourectomy + ALND	3/218 (1.4%)	0/45 (0%)	3/127 (2.4%)	0/45 (0%)	
**Radiotherapy**	215/218 (98.6%)	45/45 (100%)	126/127 (99.2%)	43/45 (95.6%)	NC
Before surgery	9/218 (4.1%)	0/45 (0%)	7/127 (5.5%)	2/45 (4.4%)	0.15
After surgery	206/218 (94.5%)	45/45 (100%)	119/127 (93.7%)	41/45 (91.1%)	
Dose (Gy)	50 (50–50)	50 (50–50)	50 (50–50)	50 (50–50)	0.78
Fractions	25 (25–25)	25 (25–25)	25 (25–25)	25 (22–25)	0.55
Overall treatment time (days)	37 (35–41)	37 (34–38)	37 (35–42)	38 (35–42)	0.21
Target area					
B or CW alone	7/216 (3.2%)	1/45 (2.2%)	5/126 (4%)	1/44 (2.3%)	0.78
B/CW + Level 2–3–4	206/216 (95.4%)	44/45 (97.8%)	120/126 (95.2%)	41/44 (93.2%)	0.59
Internal mammary node	148/212 (69.8%)	25/45 (55.6%)	95/124 (76.6%)	28/42 (66.7%)	0.026
Level 1	52/212 (24.5%)	7/45 (15.6%)	37/124 (29.8%)	8/42 (19.1%)	0.10

Data are median (IQR) or n/N (%). Percentages may not total 100 because of rounding. *p* values were calculated by using the χ^2 test, Fisher's exac*t* test, Mann–Whitney U test, or Student *t* test. HR = Hormonal Receptor. HER2 = Human Epidermoid growth factor Receptor-2. TN = Triple Negative. NC = No Computed. (F)EC-T = epirubicin with cyclophosphamide (+/- 5-Fluoro-uracil) plus docetaxel/paclitaxel. AC-T = doxorubicin with cyclophosphamide plus docetaxel/paclitaxel. TDM-1 = trastuzumab-emtansine. SLND = Sentinel Lymph Node Dissection. ALND = Axillary Lymph Node Dissection. B = Breast. CW = Chest Wall. Level 1–4 = regional lymph node areas.

Table S5. Univariate analysis of prognostic factors for overall survival (OS) stratified by triple-negative status.

		HR+ and/or HER2+, N = 218		TN, N = 99	
		**HR and 95% CI**	***p* Value**	**HR and 95% CI**	***p* Value**
Age, years (category)	<40	1		1	
	40–49	1.41 [0.38; 5.21]	0.60	0.69 [0.28; 1.72]	0.43
	50–59	1.17 [0.31; 4.40]	0.82	0.54 [0.2; 1.44]	0.22
	≥60	1.37 [0.37; 5.05]	0.64	0.71 [0.28; 1.80]	0.47
BMI, kg/m2	<25	1		1	
	≥25 <30	0.82 [0.33; 2.08]	0.68	1.13 [0.52; 2.45]	0.764
	≥30	0.82 [0.33; 2.01]	0.66	1.02 [0.47; 2.21]	0.97
Histology	Ductal	1		1	
	Lobular	0.37 [0.05; 2.70]	0.32	1.76 [0.24; 12.89]	0.58
	Other	0.63 [0.09; 4.65]	0.65	1.73 [0.41; 7.22]	0.45
SBR	1–2	1		1	
	3	1.53 [0.74; 3.19]	0.25	0.84 [0.40; 1.79]	0.65
cN	N0	1		1	
	N+	0.83 [0.36; 1.95]	0.67	1.73 [0.53; 5.65]	0.36
WHO performance status	0	1		1	
	1–2–3	1.7 [0.73; 3.99]	0.22	1.38 [0.57; 3.31]	0.48
Preoperative radiotherapy	No	1		1	
	Yes	2.38 [0.56; 10.07]	0.24	4.06 [1.77; 9.31]	0.0010
Sataloff T	TA-TB	1		1	
	TC-TD	2.73 [1.32; 5.65]	0.007	2.16 [1.12; 4.17]	0.022
Sataloff N	NA-NB	1		1	
	NC-ND	1.83 [0.81; 4.13]	0.15	7.47 [3.08; 18.12]	<0.0001

Cox proportional hazards regression analysis of overall survival. HR = hazard ratio. CI = confidence interval. HR = Hormonal Receptor. HER2 = Human Epidermoid growth factor Receptor-2. TN = Triple Negative. BMI = Body Mass Index. cN = clinical lymph Node. WHO = World Health Organization. HR = Hormonal Receptor. HER2 = Human Epidermoid growth factor Receptor-2.

Table S6. Multivariate analysis of prognostic factors for overall survival (OS) stratified by triple-negative status.

**HR+ and/or HER2+, N = 218**		**HR and 95% CI**	***p* Value**
Sataloff T	TA-TB	1	
	TC-TD	2.49 [1.11; 5.57]	0.027
Sataloff N	NA-NB	1	
	NC-ND	1.26 [0.51; 3.10]	0.621
**TN, N = 99**		**HR and 95% CI**	***p* Value**
Sataloff T	TA-TB	1	
	TC-TD	0.86 [0.42; 1.78]	0.69
Sataloff N	NA-NB	1	
	NC-ND	6.91 [2.69; 17.77]	<0.0001
Preoperative radiotherapy	No	1	
	Yes	2.14 [0.90; 5.09]	0.085

Factors with p value less than 0.1 in univariate analyses were included in multivariate Cox proportional hazards regression analysis of overall survival. HR = hazard ratio. CI = confidence interval. HR = Hormonal Receptor. HER2 = Human Epidermoid growth factor Receptor-2. TN = Triple Negative.

Figure S1. Flow chart of the patient population.

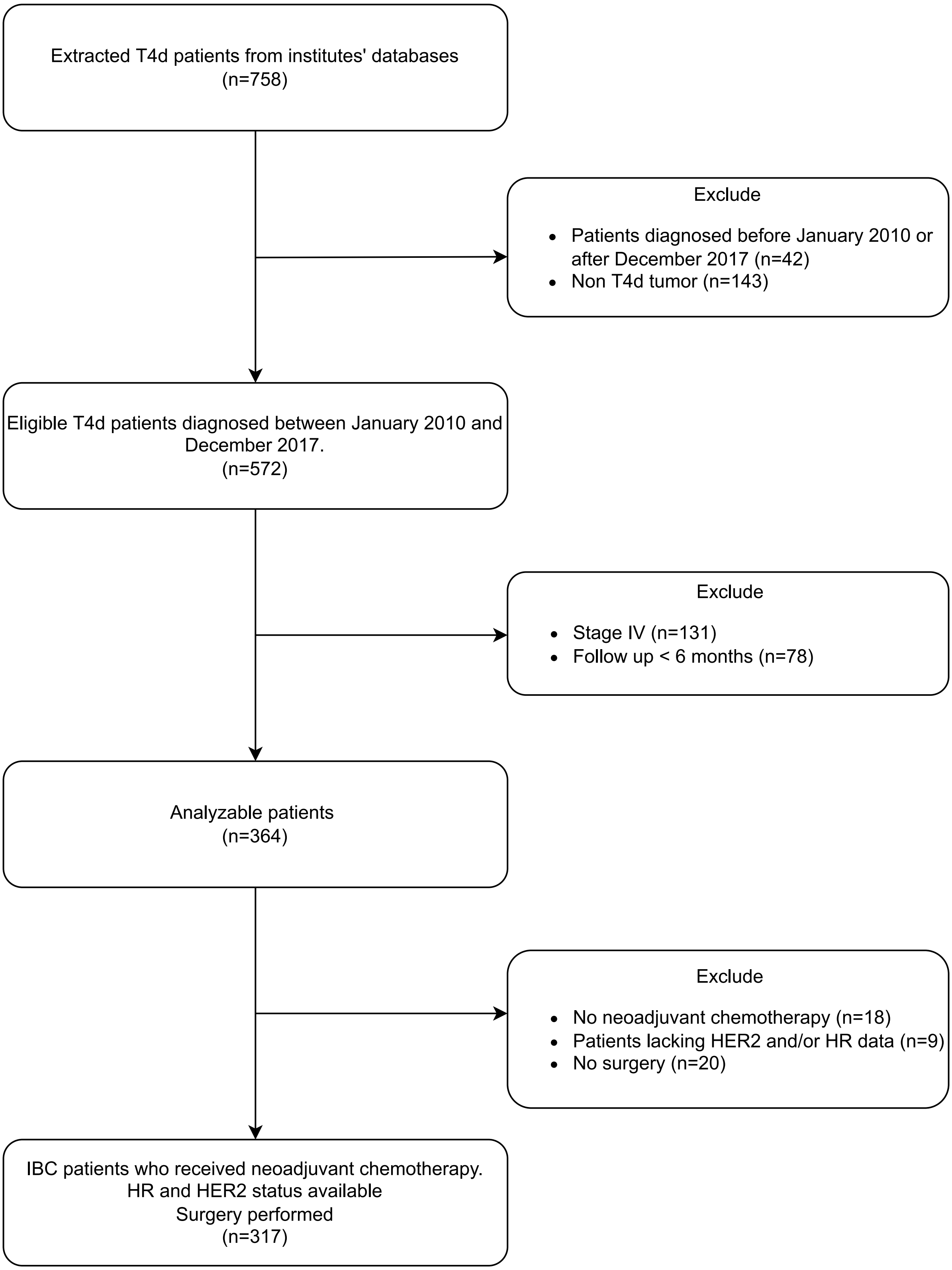

Figure S2. Flowchart of different therapeutic strategies.

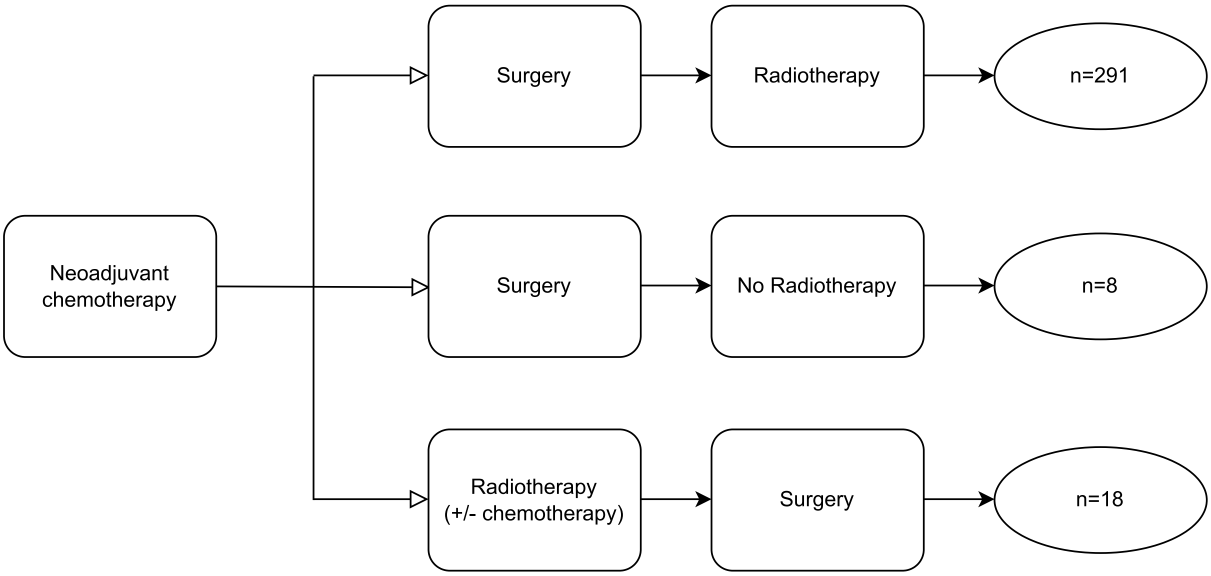

Figure S3. Association between DFS and Sataloff response by triple-negative status.

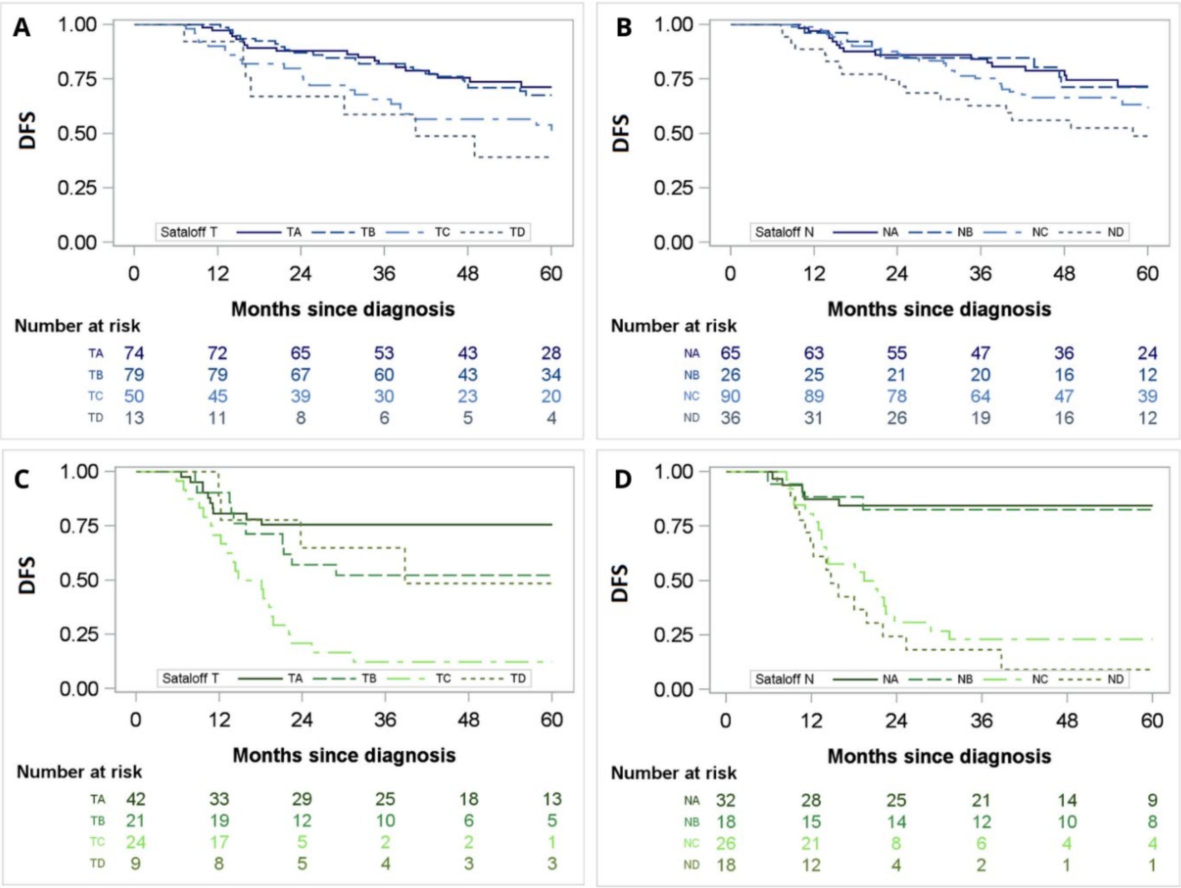

DFS = Disease Free Survival

A: Association between DFS and Sataloff T response in patients with HR+ and/or HER2+ tumours

B: Association between DFS and Sataloff N response in patients with HR+ and/or HER2+ tumours

C: Association between DFS and Sataloff T response in patients with triple negative tumours

D: Association between DFS and Sataloff N response in patients with triple negative tumours.

Figure S4. Association between OS, DMFS, LRFS, and Sataloff response by triple-negative status.

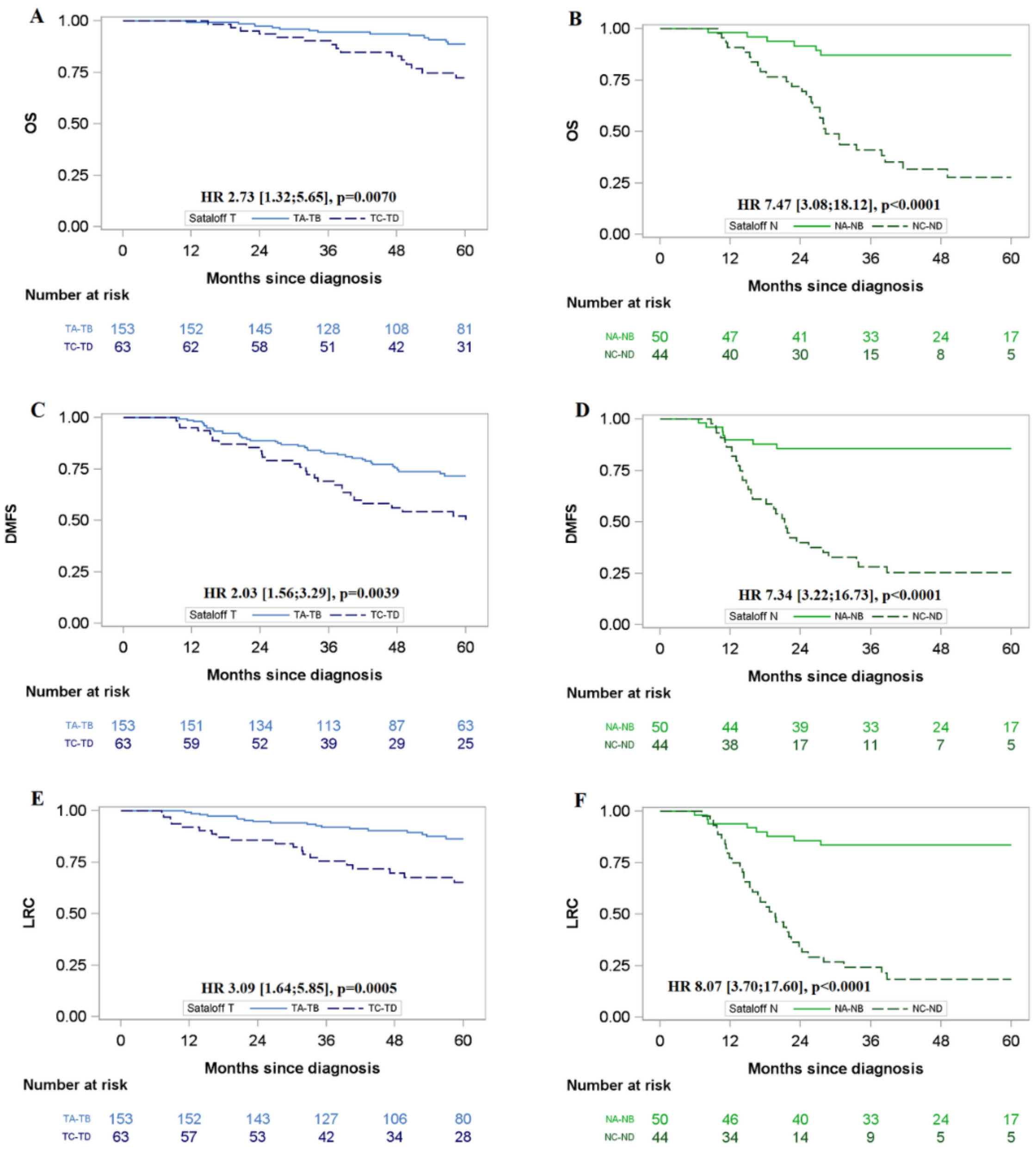

OS = overall survival. DMFS=Distant Metastasis Free Survival. LRC=locoregional recurrence-free survival.
A: Association between OS and Sataloff T response in patients with HR+ and/or HER2+ tumours
B: Association between OS and Sataloff N response in patients with triple-negative tumours
C: Association between DMFS and Sataloff T response in patients with HR+ and/or HER2+ tumours
D: Association between DMFS and Sataloff N response in patients with triple-negative tumours
E: Association between LRC and Sataloff T response in patients with HR+ and/or HER2+ tumours
F: Association between LRC and Sataloff N response in patients with triple-negative tumours.
